# Proteomic landscape of IGF-I gene transfer in osteoarthritis-derived human bone marrow stromal cells undergoing chondrogenesis

**DOI:** 10.1186/s13075-026-03919-2

**Published:** 2026-10-10

**Authors:** Patricia Quaranta, Jagadeesh K. Venkatesan, Valentina Calamia, Patricia Fernández-Puente, Selva Riva-Mendoza, Francisco J. Blanco, Magali Cucchiarini, Cristina Ruiz-Romero

**Affiliations:** 1 Grupo de Investigación en Reumatología (GIR), Instituto de Investigación Biomédica de A Coruña (INIBIC), Complexo Hospitalario Universitario de A Coruña (CHUAC), Sergas, Universidade da Coruña (UDC), As Xubias 84, A Coruña, 15006 Spain; 2 Center of Experimental Orthopaedics, Saarland University and Saarland University Medical Center, Kirrbergerstr. Bldg 37, Homburg, Saar 66421 Germany; 3 Unidad de Proteómica, Centro Interdisciplinar de Química y Biología (CICA), Universidad de A Coruña, A Coruña, 15008 Spain; 4 Grupo de Investigación en Reumatología y Salud (GIR-S), Departamento de Fisioterapia, Medicina y Ciencias Biomédicas, Facultad de Fisioterapia Universidade da Coruña (UDC), A Coruña, 15006 Spain; 5 Centro de Investigación Biomédica en Red de Bioingeniería, Biomateriales y Nanomedicina (CIBER-BBN), Madrid, 28029 Spain

**Keywords:** Bone marrow-derived adherent stromal cells, Proteomics, Chondrogenesis, Insulin-like growth factor-I, Recombinant adeno-associated virus

## Abstract

**Background:**

Osteoarthritis (OA) is a progressive joint disease marked by the breakdown of cartilage and impaired repair capacity. Gene therapy applied to human bone marrow-derived adherent stromal cells (BM-MSCs) with recombinant adeno-associated virus (rAAV) vectors represents a promising strategy for cartilage regeneration. However, the molecular mechanisms underlying its effects remain largely unexplored. In this study, we investigated the proteomic changes induced by rAAV-mediated insulin-like growth factor I (IGF-I) gene transfer during the chondrogenic differentiation of BM-MSCs derived from OA patients.

**Methods:**

BM-MSCs from OA donors were transduced with recombinant rAAV vectors encoding either IGF-I or the *lacZ* gene (β-galactosidase) as a control and cultured in three-dimensional micromass systems under chondrogenic conditions for 21 days. Histological, immunohistochemical (IHC) and immunofluorescence (IF) analyses were performed to characterize the chondrogenic phenotype of the model and to provide orthogonal validation of selected proteomic findings. Mass spectrometry-based proteomic profiling identified protein detection patterns and quantitative changes associated with IGF-I overexpression. Pathway and protein-protein interaction (PPI) analyses were carried out to explore the functional implications of differentially expressed proteins.

**Results:**

A total of 4,284 proteins were identified, of which 112 showed notable changes in detection patterns or abundance associated with IGF-I. Functional enrichment analyses showed involvement of pathways related to metabolism, endoplasmic reticulum (ER) homeostasis, apoptosis regulation, cell proliferation, glycosylation, and the endosomal sorting complex required for transport (ESCRT) machinery. Histological and IHC analyses provided supportive evidence of cartilage-related matrix deposition. Orthogonal validation by IHC and IF confirmed the IGF-I-mediated upregulation of β-catenin and exportin-1 (XPO1). Further analyses provided exploratory evidence of increased active β-catenin and predominantly extranuclear distribution of sirtuin-1, a known XPO1 substrate, in IGF-I-transduced micromasses.

**Conclusion:**

This study provides the first proteomic characterization of rAAV-mediated IGF-I gene therapy in OA-derived BM-MSCs undergoing chondrogenesis. The IGF-I gene transfer was associated with broad proteomic changes involving metabolic and protein processing pathways, and enhanced cartilage-specific matrix deposition, highlighting molecular mechanisms of potential relevance to cartilage repair in OA.

**Supplementary Information:**

The online version contains supplementary material available at https://doi.org/10.1186/s13075-026-03919-2.

## Background

 Osteoarthritis (OA) is the most prevalent rheumatic disease and a leading cause of disability worldwide. The disease is characterized by a progressive loss of articular cartilage, leading to joint dysfunction and reduced quality of life. Current treatments, including anti-inflammatory drugs and intra-articular injections of hyaluronic acid can alleviate symptoms, but none of them halt cartilage degeneration or repair cartilage defects [1]. Consequently, there is a growing interest in regenerative therapies aimed at promoting cartilage repair. Mesenchymal stem cells (MSCs) are promising candidates for this purpose, however MSC-based therapies have not consistently resulted in the formation of functional, hyaline-like repair tissue that fully integrates with the surrounding cartilage [2].

In recent years, gene therapy has emerged as an attractive approach for cartilage regeneration, enabling the controlled delivery into the joint space and sustained expression of therapeutic agents involved in cartilage repair [3]. In particular, recombinant adeno-associated virus (rAAV) vectors are capable of efficient and long-term transgene expression in vivo.

Gene transfer of therapeutic growth factors using rAAV vectors can successfully transduce articular chondrocytes within their native extracellular matrix (ECM) [4] environment. Among the different anabolic factors for cartilage repair, Insulin-like growth factor-I (IGF-I) has been shown to stimulate ECM synthesis, enhance chondrocyte proliferation and differentiation [5]. Previous studies have demonstrated the beneficial effects of rAAV-mediated IGF-I in both in vitro and in vivo models of OA [6, 7]. In particular, rAAV-mediated IGF-I expression in chondrocyte and MSC cultures increased the production of proteoglycans (PGs) and type-II collagen [8], while in vivo, intra-articular administration of therapeutic strategi proved cartilage repair and reduced OA progression in relevant animal models [9]. Although rAAV-mediated IGF-I expression has demonstrated regenerative effects in cartilage and MSC-based models, the global molecular adaptations associated with this process remain largely unexplored.

The aim of this study was to characterize the proteomic changes induced by rAAV-mediated IGF-I gene therapy in human bone marrow-derived adherent stromal cells (BM-MSCs) undergoing chondrogenic differentiation. This work seeks to provide deeper insights into the underlying molecular pathways associated with IGF-I overexpression and to explore their potential involvement in processes related to cartilage regeneration in OA.

## Methods

### Experimental design

The study was approved by the Ethical Committee of the Saarland Physicians Council (ethics application approval registration number: Ha67/12). All procedures were conducted in accordance with the Declaration of Helsinki, and informed consent was obtained from all patients prior to their inclusion in the study. Bone marrow aspirates were collected from the distal femurs of four independent OA donors undergoing total knee arthroplasty (age 63 ± 8 years) at the Department of Orthopaedic Surgery of the Saarland University Medical Center (Homburg/Saar, Germany).

The study comprised two complementary experimental components. The first was a proteomic analysis performed on BM-MSCs from three donors: Donor 1 was a 63 year-old female, Donor 2 a 66 year-old female and Donor 3 a 71 year-old male. For each donor, cells were subjected to chondrogenic differentiation under two conditions (*lacZ* and IGF-I), and two independent micromass pellets were generated per donor and condition, for a total of 12 pellets. The rAAV-*lacZ* group was included as the vector control, as it underwent identical micromass formation, transduction, and chondrogenic differentiation procedures as the rAAV-IGF-I group, thereby allowing comparisons to be attributed specifically to IGF-I overexpression rather than to vector administration. A non-transduced control group was not included due to the limited BM-MSC yield obtained from the available bone marrow aspirates. During culture, one pellet was lost due to handling during medium changes, resulting in five micromass cultures per condition and a total of 10 samples available for proteomic analysis. The second component comprised histological characterization of the in vitro model and the orthogonal validation of selected proteomic findings, performed using BM-MSCs from the fourth donor (female; 58 years-old). In this case, three independent micromass cultures were generated from this donor for each experimental condition, and processed for histological, immunohistochemical (IHC) and immunofluorescence (IF) analyses.

### Cell culture and gene transfer procedure

BM-MSCs were isolated, expanded in culture, and induced to chondrogenic differentiation according to established protocols [10]. Briefly, bone marrow aspirates were first washed with Dulbecco’s modified Eagle’s medium (DMEM) and centrifuged for 5 min at room temperature (RT) at 1,200 rpm (Eppendorf, Hamburg, Germany). Following centrifugation, the upper fatty layer was discarded, and the resulting pellet was resuspended in a 1:1 mixture of Red Blood Cell Lysing Buffer and DMEM. After further washing steps, the recovered cell fraction was resuspended in DMEM supplemented with 10% fetal bovine serum (FBS), 100 U/mL penicillin, and 100 µg/mL streptomycin (growth medium). Cells were plated in T75 flasks and incubated at 37 °C in a humidified atmosphere containing 5% CO_2_. After 24 h, non-adherent cells were removed by replacing the culture medium. The medium was subsequently changed every 2–3 days. Upon reaching confluence, cells were detached at passage 1 for subsequent experiments. Cell viability was assessed by Trypan blue exclusion and was ≥95% in all cultures included in the study.

Recombinant adeno-associated viral vectors were based on the parental AAV2 genomic clone pSSV9 and were packaged as conventional single-stranded rAAV2 vectors [11]. The rAAV-*lacZ* construct contains the *lacZ* gene coding for β-galactosidase (β-gal) under the control of the cytomegalovirus immediate-early (CMV-IE) promoter. The rAAV-IGF-I construct carries a 536-bp cDNA coding for human IGF-I and is also regulated by the CMV-IE promoter. Vector production was performed in adenovirus 5-transformed HEK293 cells with the pAd8 helper plasmid to provide the required helper and packaging functions [12]. Vector preparations were purified by dialysis with 10-kDa molecular-weight-cutoff dialysis cassettes (Thermo Scientific, cat. no. 66380) against 250 mL of 10% sucrose for 24 h at 4 °C. Vector titres were determined by real-time PCR and expressed as transgene copies per milliliter (approximately 10^10^ transgene copies/mL). A total of 8 × 10^5^ transgene copies was applied to 2 × 10^5^cells, corresponding to a multiplicity of infection (MOI) of 4 transgene copies/cell. Transduction efficiency was not quantified individually for each donor in the present study. However, the same procedure has been reported to achieve transduction efficiencies of up to 81% in BM-MSC micromass cultures using this experimental system [12]. Micromass cultures were generated with 2 × 10^5^ BM-MSCs. Following micromass formation, BM-MSCs were transduced with rAAV vectors (40 µL each vector, corresponding to 8 × 10^5^ functional recombinant viral particles), according to established protocols [13]. The BM-MSC micromasses chondrogenic differentiation was initiated 24 h after the transduction and was carried out over 21 days in a defined chondrogenic medium composed of high-glucose DMEM (4.5 g/L), penicillin/streptomycin, 6.25 µg/mL insulin, 6.25 µg/mL transferrin, 6.25 µL/mL selenous acid, 5.35 µL/mL linoleic acid, 1.25 µg/mL bovine serum albumin, 1 mM sodium pyruvate, 37.5 µg/mL ascorbate 2-phosphate, 10^− 7^ M dexamethasone, and 10 ng/mL transforming growth factor beta 3 (TGF-β3).

### Sample preparation for proteomic analysis by mass spectrometry (MS)

After transduction and 21 days of chondrogenic differentiation, the micromasses were washed twice and placed 24 h in serum-free medium. Then, supernatants were discarded and the micromasses were frozen at -80 °C. For protein extraction, the micromasses were resuspended by vortexing in 30 µL of 6 M urea/2 M thiourea/25 mM ammonium bicarbonate buffer and 10 µl H_2_O Optima™ liquid chromatography-mass spectrometry (LC/MS) grade. Then, samples were sonicated and centrifuged for 5 min at 4 °C and 12,000 rpm, and the supernatants were collected. Protein concentration was determined by a modified Bradford protocol and sodium dodecyl sulfate-polyacrylamide gel electrophoresis (SDS-PAGE, 10% Acrylamide) with silver stain.

For protein digestion, 5 µg of each sample were reduced with 10 mM dithiothreitol (DTT) for 1 h at 37 °C. Subsequently, cysteines were blocked by alkylation with 50 mM iodoacetamide (IA) for 45 min in the dark. The samples were then diluted with 25 mM ammonium bicarbonate to lower the final urea/thiourea concentration to 1 M and trypsin (Promega Corporation) was added at a 1:40 ratio (enzyme: protein). The enzymatic digestion was stopped after 16 h at 37 °C by decreasing the pH of the reaction down to pH = 2 with trifluoroacetic acid (TFA). Tryptic peptides were cleaned up using in-house made StageTips (2 M Empore™ Octadecyl C18 Extraction Disk, 47 mm, Sigma Aldrich). Finally, the tryptic peptides were vacuum-dried in a SpeedVac concentrator (Thermo Fisher Scientific, Massachusetts, U.S.). The peptide mixtures were reconstituted in 10 µL with 2% acetonitrile (ACN) and 0.1% formic acid (FA).

### MS proteomic analysis

The analysis was carried out following a shotgun label-free proteomic strategy with a high-resolution time-of-flight (TOF) mass spectrometer. To this end, the peptide mixtures (200 ng) were loaded in a nanoElute (Bruker) nano-flow LC coupled to a Trapped ion mobility spectrometry-time-of-flight instrument (timsTOF Pro, Bruker, Karlsruhe, Germany) with a CaptiveSpray ion source (Bruker). LC was performed at 50 °C and with a constant flow of 500 nL/min on a reversed-phase column (15 cm x 75 μm i.d.) with a pulled emitter tip, packed with 1.9 μm C18-coated porous silica beads (Dr. Maisch, Ammerbuch-Entringen). Chromatographic separation was carried out using a linear gradient of 2–35% buffer B (100% ACN and 0.1% FA) over 40 min. After electrospray ionization, peptides were analysed in data-independent mode (DIA) with the Parallel Accumulation-Serial Fragmentation (PASEF) enabled. To ensure optimal instrument performance, data quality, and reproducibility throughout LC-MS/MS acquisition, the system was calibrated prior to analysis according to the manufacturer’s instructions. A HeLa cell extract (Thermo Scientific™ Pierce™ HeLa Protein Digest Standard, PI88329) was injected at the beginning and end of the analytical run to monitor system stability, and two blank runs were included between samples to minimize carryover.

### MS data analysis

Following MS acquisition, raw files were processed with DIA-NN (version 1.8.1) with a library-free approach. The human UniProt canonical proteome (Homo sapiens taxonomy; UniProt Consortium, 2019) was used as the reference database. An initial spectral library was automatically generated and refined by DIA-NN based on the dataset at a global q-value threshold of 0.01, and the refined library was subsequently used for final data processing. Peptide precursor and protein group identifications were controlled at a false discovery rate (FDR) of 1%. Precursor and fragment mass tolerances were automatically optimized by DIA-NN. Retention time-dependent, median-based normalization across runs was applied using the “robust LC (high precision)” quantification setting. Protein quantification was performed at the protein group level based on precursor intensities.

### Statistical analysis

Statistical analysis and graphical representations were performed using R version 4.0.0 (R Core Team, 2020) on the Protein Group file from DIA-NN software. A quality control procedure to minimize false positive results was conducted. Proteins remaining from the analysis were identified with ≥ 2 unique peptides and quantified in ≥ 60% of the samples. The subset was first screened for protein detection patterns using chi-squared analysis as an exploratory tool (nominal *p* ≤ 0.05), before the filter of missing values (MV) was applied. Prior to the differential expression analysis, data preprocessing was performed. Protein intensity data were normalized using the log2 scale. MV were imputed through random draws from a left-shifted Gaussian distribution, centered at low-intensity values to approximate signals below the detection limit. Protein-wise linear models combined with empirical Bayes statistics were applied to identify differentially abundant proteins with the limma package in R/Bioconductor. Donor identity was included as a blocking factor to account for the paired experimental design and the presence of replicate cultures derived from the same donor. Differential abundance was evaluated for each pairwise comparison between experimental conditions. A cutoff of the adjusted *p* ≤ 0.05 (Benjamini-Hochberg method), along with a |fold change| ≥ 1.5 was applied to determine significantly regulated proteins in each pairwise comparison.

### Pathway and protein-protein interaction enrichment analysis

Pathway and process enrichment was conducted in Metascape (v 3.5) [14] following a variety of ontology sources: Kyoto Encyclopedia of Genes and Genomes (KEGG) Pathway [15], Gene Ontology (GO) Biological Processes, Reactome Gene Sets, Canonical Pathways, CORUM, WikiPathways, and PANTHER (Protein analysis through evolutionary relationships) Pathway. *p*-values were calculated based on the cumulative hypergeometric distribution and q-values were computed using the Benjamini-Hochberg procedure to account for multiple testings. Terms with a *p* < 0.01, a minimum count of 3, and an enrichment factor > 1.5 (where the enrichment factor compares observed counts to expected counts) were compiled and organized into clusters, based on their membership similarities.

Protein-protein interaction (PPI) enrichment analysis was carried out in Metascape (v 3.5) with the following databases: STRING (Search Tool for the Retrieval of Interacting Genes/Proteins), BioGrid (Biological General Repository for Interaction Datasets), OmniPath, InWeb_IM. The Molecular Complex Detection (MCODE) algorithm was applied to identify densely connected network components. Pathway and process enrichment analysis was then applied to each MCODE component independently, retaining the three top-scoring terms by *p* as the functional description of the corresponding components.

### Histological and immunohistochemical analyses

Micromass pellets were transduced with rAAV vectors and induced to undergo chondrogenic differentiation as described above. After 21 days, pellets were fixed in 4% formalin, dehydrated through graded alcohols, embedded in paraffin, and sectioned at 3 μm thickness. Histological evaluation was performed using hematoxylin and eosin, and Toluidine Blue staining to assess glycosaminoglycan (GAG) content.

IHC was performed to assess the expression of IGF-I, type II collagen (COL-II), Exportin 1 (XPO1/CRM1), Sirtuin 1 (SIRT1), β-catenin and the active form of β-catenin. Sections were incubated with the following primary antibodies: anti-IGF-I (goat; R&D bio-techne, Wiesbaden, Germany; 1:25 dilution), anti-COL-II (mouse; DSHB, Iowa City, IA, USA; used undiluted), anti-CRM1 (rabbit; Abcam; 1:50 dilution), anti-SIRT1 (rabbit, Abcam, Cambridge, UK; 1:100 dilution), anti-β-catenin (rabbit; Cell Signaling Technology, MA, USA; 1:25 dilution) and the active form of β-catenin was evaluated by mouse monoclonal antibody specifically recognizing β-catenin dephosphorylated at Ser37 or Thr41 (mouse, Sigma-Aldrich, Saint Louis, Missouri, USA; dilution 1:100). Appropriate biotinylated secondary antibodies were then applied.

In addition, the active form of β-catenin was evaluated by IF (mouse, Sigma-Aldrich, Saint Louis, Missouri, USA; dilution 1:100) and anti-SIRT1 (rabbit, Abcam, Cambridge, UK; 1:100 dilution). Following incubation with an appropriate fluorophore-conjugated secondary antibody, nuclei were counterstained with DAPI.

Histological and IHC sections were examined with an Olympus BX45 microscope, which was also used for epifluorescence imaging. For each condition, three independently prepared micromass cultures derived from the same donor were analysed. Images encompassing the complete micromass section were acquired at 4× magnification. The entire micromass area was manually delineated and used as the region of interest. For Toluidine Blue staining and the quantitative IHC analyses, a staining threshold was defined for each marker and subsequently kept constant across both experimental conditions and all culture replicates. Positive staining was expressed as the percentage of the total micromass area using Fiji software. Image acquisition and quantitative analysis were performed by an investigator blinded to the experimental condition. Data are presented as the mean ± SD of three micromass cultures per condition. Paired comparisons were performed using a paired *t*-test, with statistical significance set at *p* < 0.05.

## Results

### Characterization of the chondrogenic BM-MSCs model and IGF-I overexpression

**Fig. 1:**
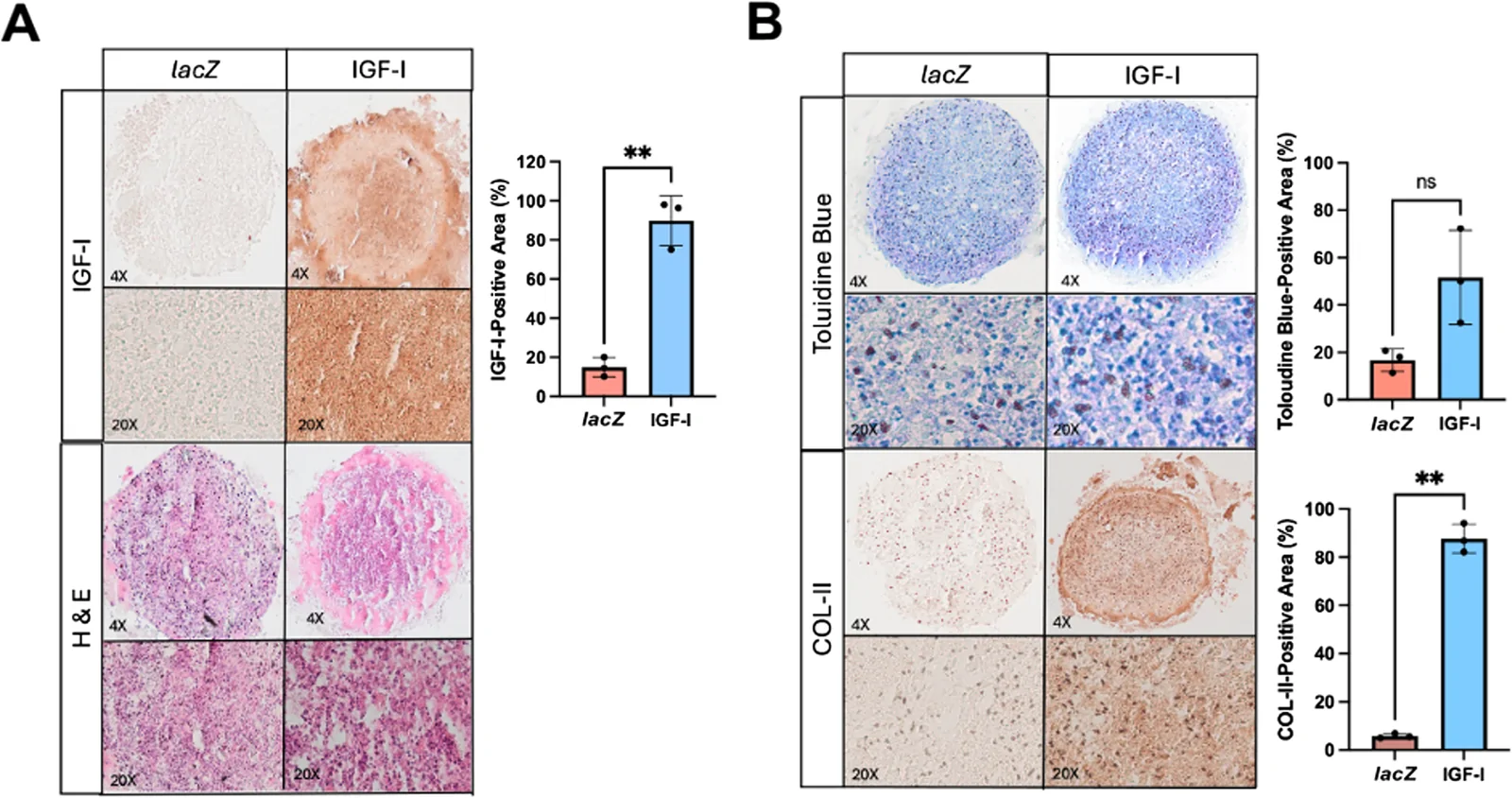
Phenotypic characterization of chondrogenic differentiation in rAAV-IGF-I-transduced BM-MSC micromass cultures. Representative histological and IHC staining of micromass cultures transduced with *lacZ* (control) or IGF-I vectors after 21 days of chondrogenic differentiation. **A** IGF-I immunostaining and hematoxylin and eosin (H&E) staining, with quantification of the IGF-I-positive area (%). **B** Toluidine Blue, and collagen type II (COL-II) staining, with quantification of the corresponding positive staining areas (%). Images are shown at 4× and 20× magnification. Data are presented as mean ± SD (*n* = 3). **p < 0.01; ns, not significant

BM-MSC micromasses were transduced with rAAV vectors encoding either IGF-I or *lacZ*, and subsequently induced to undergo chondrogenic differentiation for 21 days, following a previously standardized procedure [12]. Histological and IHC analyses were performed to characterize this model (*n* = 3). As shown in Fig. 1A, hematoxylin and eosin staining revealed a preserved micromass architecture in both experimental conditions, with no evident morphological alterations. IHC analysis confirmed increased IGF-I expression within the micromasses transduced with rAAV-IGF-I compared to *lacZ*-transduced controls, with a significantly higher IGF-I-positive area (89.8 ± 12.6% vs. 14.9 ± 4.9%, *p* < 0.01), consistent with successful transgene expression.

As previously reported for this model [12], the chondrogenic phenotype was further supported by evaluating cartilage ECM-related markers. Toluidine Blue staining (Fig. 1B) demonstrated the presence of GAG-rich ECM in micromass cultures, indicating baseline matrix production under chondrogenic conditions. A numerical increase in GAG deposition was observed in rAAV-IGF-I-transduced micromasses compared to controls (51.7 ± 19.9% vs. 16.7 ± 4.8%), although this difference did not reach statistical significance. In contrast, COL-II immunostaining exhibited a markedly stronger signal in rAAV-IGF-I-transduced micromasses compared with *lacZ* controls (87.6 ± 6.0% vs. 5.8 ± 0.9%, *p* < 0.01) (Fig. 1B), supporting an IGF-I-associated enhancement of cartilage-specific matrix deposition. Together, these results confirmed the chondrogenic competence of the model and the IGF-I- associated enhancement of cartilage-specific matrix deposition.

### Proteomic analysis of IGF-I-transduced BM-MSCs

To investigate the molecular mechanisms underlying the effects of rAAV-mediated IGF-I overexpression during chondrogenic differentiation, a comprehensive proteomic analysis was carried out on 10 micromass cultures after 21 days of differentiation, comprising five rAAV-IGF-I-transduced and five *lacZ*-transduced samples.

**Fig. 2:**
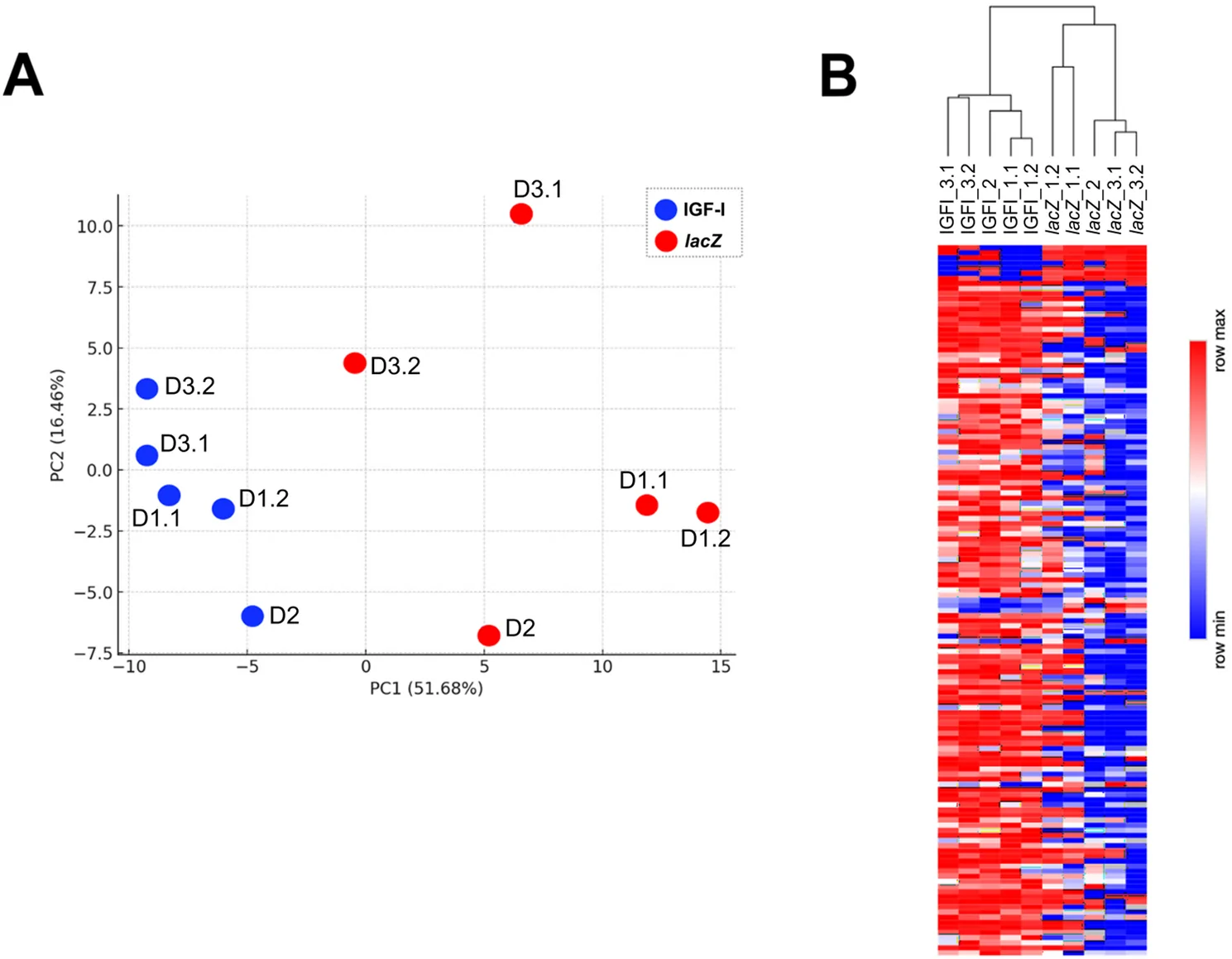
Proteomic profiling of BM-MSCs transduced with IGF-I. **A** Principal component analysis (PCA) plot comparing the proteomic profiles of samples from the IGF-I (blue) and *lacZ* (red) groups. **B** Heatmap displaying the differences in protein abundance between the IGF-I and *lacZ* groups, with red indicating higher abundance and blue representing lower abundance

A total of 4,284 proteins were identified in the micromass proteome. An unsupervised principal component analysis (PCA) was performed to explore differences between the experimental groups. As shown in Fig. 2A, the IGF-I group was clearly separated from the *lacZ* control group, with PC1 accounting for 51.68% of the variance. Consistent with the PCA results, hierarchical clustering revealed two different clusters according to the condition groups (Fig. 2B), indicating substantial differences in protein expression profile between rAAV-IGF-I- and *lacZ-*transduced micromasses.

### Exploratory analysis of protein detection patterns associated with IGF-I

**Fig. 3:**
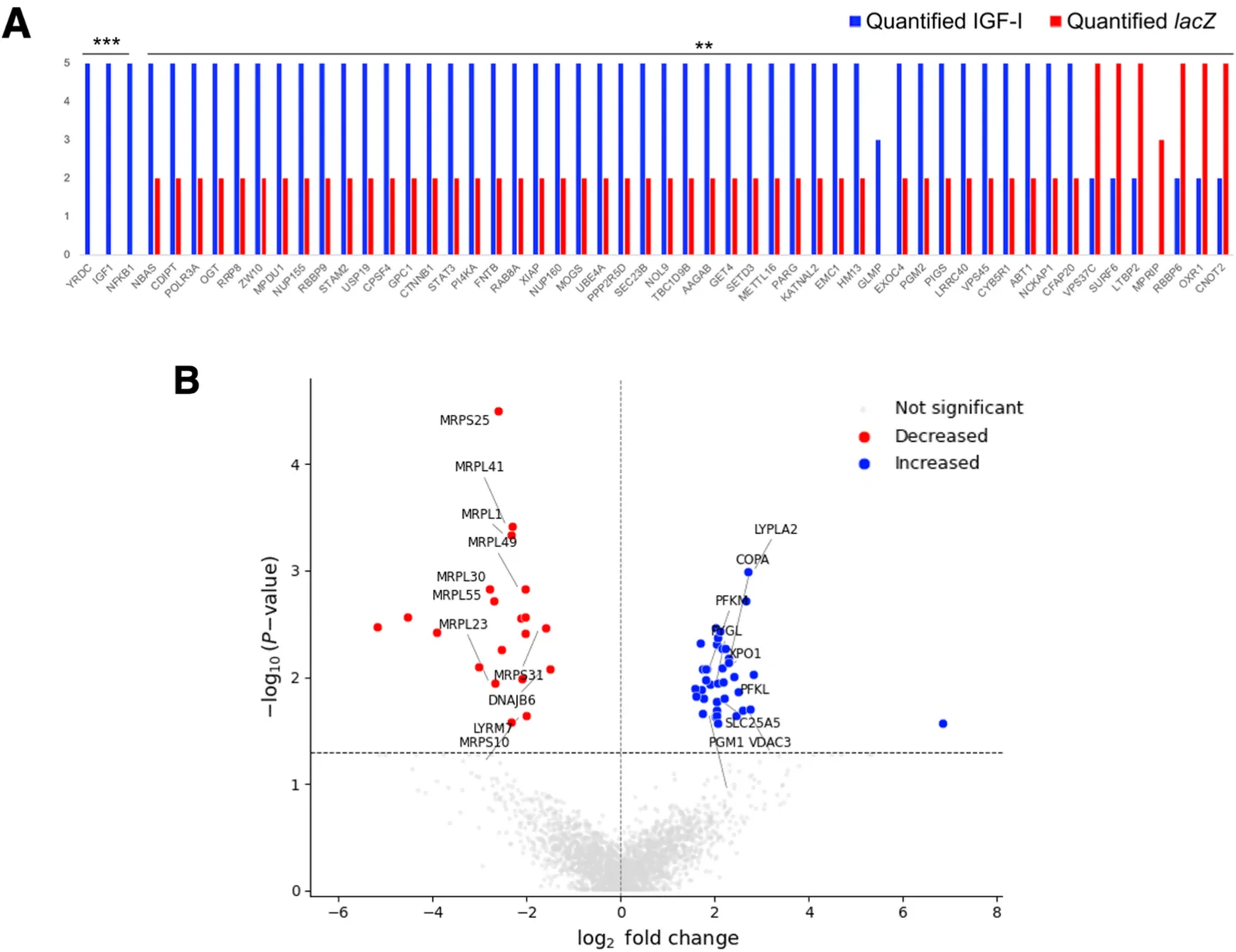
Proteomic changes induced by IGF-I treatment in BM-MSCs. **A** Exploratory analysis of protein detection patterns between rAAV-IGF-I-transduced and rAAV-*lacZ* control micromasses. The displayed proteins showed nominal differences in detection frequency in the chi-squared screening (**p ≤ 0.05, ***p ≤ 0.01). Bars represent the number of samples in which each protein was detected, with IGF-I samples shown in blue and *lacZ* controls in red. **B** Volcano plot of differential protein expression analysis. Proteins with |fold change| ≥ 1.5 and adjusted *p* ≤ 0.05 were considered significantly differentially expressed. Proteins upregulated by IGF-I gene transfer are shown in red, and downregulated proteins in blue

After an initial filter for unique peptides ≥ 2, 3,739 proteins remained and are listed in Supplementary Table (1) An exploratory presence/absence analysis was performed with chi-squared test. This analysis identified 54 proteins with nominal differences in the detection frequency (*p* ≤ 0.05), listed in Supplementary Table (2) Of these, 47 proteins were quantified in the majority of IGF-I samples but were not detected in most of the *lacZ* controls (Fig. 3A), including IGF-I, Threonyl Carbamoyl-AMP synthase (YRDC), Nuclear factor NF-kappa-B p105 subunit (NFKB1), β-catenin (CTNNB1), and Glycosylated lysosomal membrane protein (GLMP). Conversely, 7 proteins were detected in the control group but not in most IGF-I samples, suggesting a downregulation by IGF-I. Among them, Myosin phosphatase Rho-interacting protein (MPRIP) was not detected in any of the IGF-I samples. Given the limited sample size and the exploratory nature of this analysis, these results should be interpreted as hypothesis-generating observations rather than statistically confirmed differences, and were used as such for subsequent pathway enrichment analysis.

### Evaluation of quantitative differences in the proteomic profile induced by IGF-I

The proteome was then filtered to remove those proteins showing ≥ 40% of MV, in order to focus on those that are consistently detected and explore quantitative changes on them. The remaining 2,652 proteins were analysed for differential expression combining linear models with empirical Bayes statistics. Fifty-eight proteins exhibited significant differential expression (|fold change| ≥ 1.5 and adjusted *p* ≤ 0.05) between IGF-I and *lacZ*-transduced micromasses (Fig. 3B) and are listed in Supplementary Table 3. Of these, 38 proteins showed a significant upregulation in samples with the chondrogenic factor IGF-I compared with the control (fold change from 6.85 to 1.57), including Apolipoprotein E (APOE), Fatty aldehyde dehydrogenase (ALDH3A2) and GTP-binding protein SAR1a (SAR1A).

Conversely, 20 proteins showed significant downregulation in the IGF-I group (fold change from − 5.18 to -1.50 and adjusted *p* ≤ 0.05). These proteins are mostly associated with mitochondrial translation, including Complement component 1 Q subcomponent-binding protein (C1QBP), Small ribosomal subunit proteins mS25 (MRPS25) and uS10m (MRPS10), and Large ribosomal subunit protein uL30m (MRPL30).

### Pathway and protein-protein interaction enrichment analysis

**Fig. 4:**
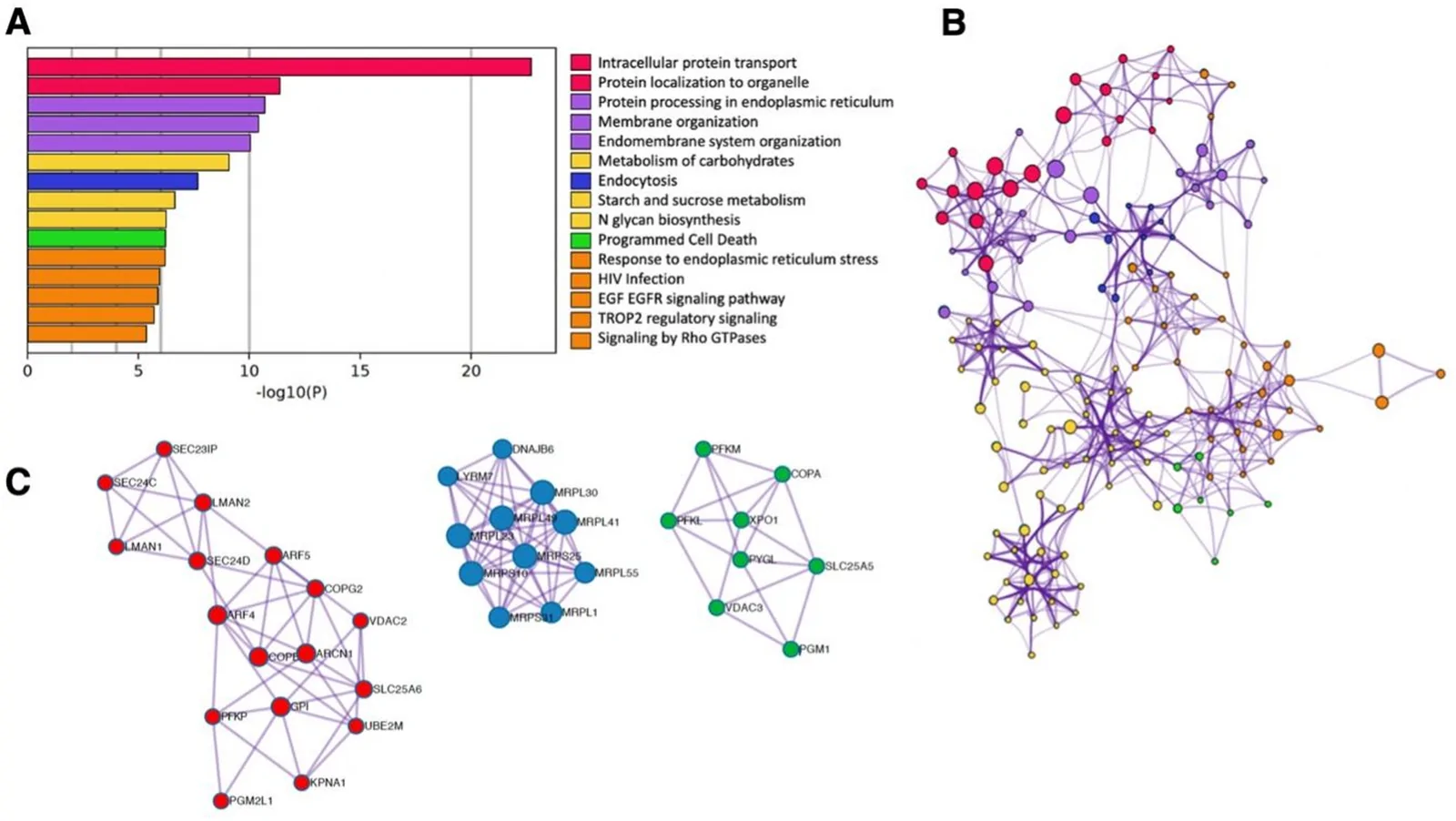
Functional enrichment and protein–protein interaction (PPI) analyses of IGF-I-regulated proteins. **A** Metascape enrichment network visualization displaying the 15 most significant clusters, grouped into six overarching functional pathways: Transport and Localization (pink), Protein Processing and Organization (purple), Metabolism (yellow), Migration (blue), Apoptosis (green), and Proliferation/Survival (red). **B** Network representation illustrating protein interactions within the six overarching pathways identified in the enrichment analysis. **C** PPI enrichment analysis using the Molecular Complex Detection (MCODE) algorithm, identifying three major functional clusters: MCODE_1 (red), associated with Golgi vesicle transport and endoplasmic reticulum (ER)-to-Golgi trafficking; MCODE_2 (blue), linked to mitochondrial ribosomal function and translation, and MCODE_3 (green), related to carbohydrate metabolism

To elucidate the functional pathways related to the observed proteomic changes caused by IGF-I, a pathway and process enrichment analysis was performed on the 112 significant proteins, including both detection frequency and quantitative changes. This analysis led to the identification of 15 clusters with the most significant p-values, which are illustrated in Fig. 4A. These clusters were involved in 6 overarching pathways: protein processing and organization, intracellular transport and localization, apoptosis, proliferation/survival, metabolism, and migration (Fig. 4B). The complete list of proteins included in each cluster is shown in Supplementary Table 4.

To further explore functional relationships, PPI enrichment analysis was performed on these 112 proteins, identifying three densely connected network components (Fig. 4C). The top enriched terms associated with each MCODE component are summarized in Table 1. The first cluster is associated with processes related to intracellular transport, such as ER to Golgi anterograde and vesicle-mediated transport. The second cluster is associated with mitochondrial-related processes, including the 55 S mitochondrial ribosome and mitochondrial translation initiation and elongation. Proteins within this mitochondrial cluster showed decreased abundance in IGF-I-transduced micromasses, including Small ribosomal subunit proteins uS10m (MRPS10) and mS25 (MRPS25), and Large ribosomal subunit protein uL30m (MRPL30). The third cluster is related to metabolic processes, including carbohydrate catabolism, the pentose phosphate pathway, and galactose metabolism.

**Table 1:** Results from the PPI network analysis. This analysis was performed on the 112 proteins significantly modulated in BM-MSCs by IGF-I treatment, using the MCODE algorithm to identify densely connected network components

MCODE	GO	Description	Log10(P)
MCODE 1	R-HSA-199977	ER to Golgi anterograde transport	−18.8
MCODE 1	R-HSA-948021	Transport to the Golgi and subsequent modification	−18.0
MCODE 1	GO:0006888	Endoplasmic reticulum-to-Golgi vesicle-mediated transport	−17.0
MCODE 2	CORUM:320	55 S ribosome, mitochondrial	−21.9
MCODE 2	R-HSA-5389840	Mitochondrial translation elongation	−21.4
MCODE 2	R-HSA-5368286	Mitochondrial translation initiation	−21.4
MCODE 3	GO:0016052	Carbohydrate catabolic process	−8.0
MCODE 3	hsa00030	Pentose phosphate pathway	−7.3
MCODE 3	hsa00052	Galactose metabolism	−7.2

BM-MSCs (bone marrow-derived adherent stromal cells), PPI (Protein-protein interaction), IGF-I (Insulin-like growth factor I), MCODE (Molecular Complex Detection), Log10(P) (logarithm base 10 (*p*))

### Orthogonal validation of the IGF-I-mediated regulation of β-catenin and exportin-1

To provide orthogonal validation of selected proteomic findings, two proteins with significant regulation in the mass spectrometry analysis were selected for independent evaluation by IHC: β-catenin (CTNNB1), a key effector of the Wnt signalling pathway with established relevance to IGF-I signalling and cartilage biology [16, 17], which was identified in the protein detection patterns dataset; and XPO1, a nuclear export receptor implicated in chondrocyte survival and cartilage homeostasis [18, 19], identified in the quantitative analysis.

**Fig. 5:**
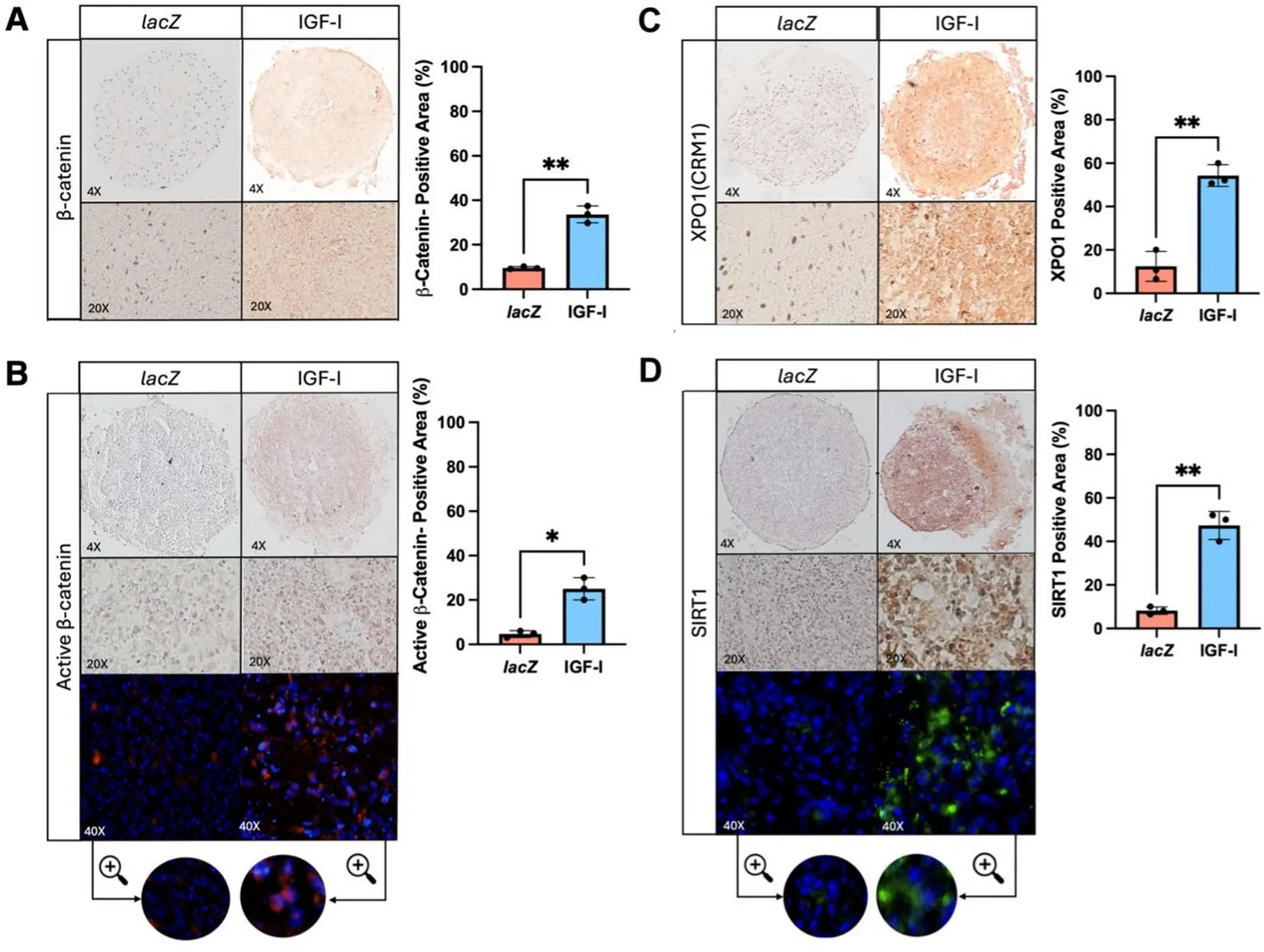
Validation of IGF-I-associated upregulation of β-catenin and XPO1 in rAAV-IGF-I-transduced BM-MSC micromass cultures. Representative IHC and IF images of micromass cultures transduced with *lacZ* (control) or rAAV-IGF-I vectors after 21 days of chondrogenic differentiation. **A** Total β-catenin expression evaluated by IHC. **B** Active β-catenin (dephosphorylated at Ser37/Thr41) evaluated by IHC and IF. **C** XPO1/CRM1 expression evaluated by IHC. **D** SIRT1 expression and subcellular distribution evaluated by IHC and IF. Nuclei were counterstained with DAPI in the immunofluorescence images. Quantification of the positive staining area is shown in the corresponding bar plots. Data are presented as mean ± SD from three independently prepared micromass cultures derived from the same donor. Statistical comparisons were performed using a paired *t*-test. **p < 0.01; *p < 0.05

β-catenin was identified with four unique peptides in all IGF-I-transduced samples but only in two control samples (*p* = 0.038). IHC analysis showed a significantly higher β-catenin-positive area in IGF-I-transduced samples compared to controls (33.7 ± 3.8% vs. 9.6 ± 0.6%, *p* < 0.01) (Fig. 5A). Staining with an antibody specific for β-catenin dephosphorylated at Ser37 or Thr41 confirmed an increase in active β-catenin in rAAV-IGF-I-transduced micromasses compared with rAAV-*lacZ* controls (25.00 ± 5.0% vs. 4.67 ± 1.53%, *p* = 0.024). Active β-catenin immunoreactivity showed both nuclear and extranuclear distribution, with visually appreciable nuclear staining as shown in the IF (Fig. 5B). Together, these findings demonstrated an IGF-I-associated increase in both total and active β-catenin.

XPO1 was significantly upregulated in IGF-I-transduced micromasses (FC = 2.24, adjusted *p* = 0.024). Immunostaining (Fig. 5C) revealed a significant increase in XPO1-positive area in the rAAV-IGF-I-transduced micromasses compared with *lacZ* controls (54.3 ± 5.0% vs. 12.4 ± 7.0%, *p* < 0.01), consistent with its identification in the proteomic analysis. To assess the functional relevance of XPO1 upregulation, SIRT1 was evaluated as a downstream readout of XPO1-mediated nuclear export. SIRT1 is a nucleocytoplasmic shuttling protein whose export from the nucleus is mediated by XPO1/CRM1 [20]. Immunostaining analysis showed markedly increased SIRT1 staining in rAAV-IGF-I-transduced micromasses compared with rAAV-*lacZ* controls (47.33 ± 6.43% vs. 8.19 ± 1.72%, *p* < 0.01), with a predominantly extranuclear distribution (Fig. 5D). Together with the proteomic and IHC results for XPO1, this finding supports increased activity of the XPO1-SIRT1 nucleocytoplasmic transport axis following IGF-I gene transfer.

These results provide independent validation of two proteomic findings and support the IGF-I-associated modulation of proteins involved in intracellular signalling and transport during chondrogenic differentiation.

## Discussion

OA is a progressive joint disease characterized by ECM degradation, loss of proteoglycans, and disruption of type II collagen, ultimately leading to cartilage damage and joint dysfunction [21]. Within this context, IGF-I has emerged as a key regulator of cartilage homeostasis, promoting chondrocyte proliferation, survival, and ECM synthesis while inhibiting chondrocyte apoptosis [13, 22].

In the present study, we investigated the effects of rAAV-mediated IGF-I overexpression during the chondrogenic differentiation of BM-MSCs from OA patients using a proteomic approach. IGF-I overexpression, confirmed by IHC and proteomic analysis, was associated with broad proteomic changes: upregulated proteins were predominantly involved in metabolic processes, protein processing, and intracellular transport, while downregulated proteins were mainly related to mitochondrial functions. This pattern is consistent with a shift toward an anabolic and biosynthetically active cellular state, which may be particularly relevant during chondrogenic differentiation, a process that requires extensive metabolic adaptation to support ECM production and tissue formation [23].

Pathway and PPI analyses highlighted glycolysis and carbohydrate metabolism as significantly enriched in IGF-I-transduced micromasses. This enrichment, together with the relative decrease in mitochondrial-associated proteins, indicates that the IGF-I-associated proteomic changes involve glycolysis- and carbohydrate metabolism-related pathways, a feature commonly associated with proliferating or differentiating cells [24]. Given that metabolic dysregulation is recognized as a contributor to OA pathogenesis [25], these findings suggest a potential association between IGF-I gene transfer and pathways related to carbohydrate metabolism, though functional confirmation will require dedicated metabolic assays in future studies.

The identification of glycosylation-related proteins, including O-linked β-N-acetylglucosamine transferase (OGT) [26, 27], together with enrichment of N-glycosylation pathways [28] and detection of extracellular glycoproteins such as biglycan (BGN) and glypicans (GPCs), suggests that IGF-I may influence ECM organization through coordinated regulation of protein synthesis and post-translational processing [29]. Proteoglycans are essential components of articular cartilage and their altered composition is a hallmark of OA [30], thus the increased abundance of these proteins in IGF-I-transduced micromasses is of potential relevance to cartilage homeostasis.

In addition to metabolic and ECM-related changes, our data suggest modulation of ER homeostasis and protein processing pathways. ER homeostasis is central for protein folding and adaptation to increased biosynthetic demand [31, 32], and IGF-I has been reported to protect cells from ER stress-induced apoptosis through activation of PI3K/AKT and MAPK signalling [33, 34]. The enrichment of ER-related proteins in our dataset is consistent with this role, though the functional significance of these changes in the context of chondrogenic differentiation remains to be established.

β-catenin, a central effector of the canonical Wnt signalling pathway [16], was identified in the exploratory protein-detection analysis. This observation was supported by increased total β-catenin expression and enhanced immunoreactivity of its active form in rAAV-IGF-I-transduced micromasses. A functional interplay between IGF-I and Wnt/β-catenin signalling has recently been highlighted in OA, with IGF-I identified as a critical mediator of Wnt-driven joint pathology [17]. Additionally, Wnt/β-catenin signalling has been implicated in progenitor-like chondrocyte populations associated with cartilage repair [35]. Together, these findings support IGF-I-associated modulation of β-catenin signalling during chondrogenic differentiation. However, given the dose-dependent and context-specific effects of Wnt/β-catenin signalling on chondrocyte homeostasis [36], the functional consequences of the observed increase in active β-catenin require further mechanistic investigation.

Consistent with the enrichment of intracellular transport-related pathways, XPO1, the principal nuclear export receptor [37, 38], was significantly upregulated in IGF-I-transduced micromasses and independently validated by IHC, providing orthogonal support for this proteomic finding. XPO1 has been implicated in the regulation of chondrogenesis and chondrocyte survival through control of the subcellular localization of cartilage homeostasis-related proteins such as SIRT1 [19]. SIRT1 undergoes XPO1-dependent nucleocytoplasmic shuttling, while IGF-1R/PI3K signalling has been reported to promote the stability of cytoplasmic SIRT1 [20, 39]. In line with these mechanisms, IHC analysis showed increased SIRT1 staining in rAAV-IGF-I-transduced micromasses with a predominantly extranuclear distribution, supporting IGF-I-associated modulation of the XPO1-SIRT1 nucleocytoplasmic transport axis. Interestingly, pharmacological inhibition of XPO1 with KPT-330 has recently been reported to attenuate OA progression by suppressing subchondral bone osteoclastogenesis through modulation of NF-κB and MAPK signalling [40], highlighting the broader relevance of XPO1 in OA-related processes, albeit in a different cellular context. The precise functional implications of increased XPO1 expression during chondrogenic differentiation warrant further investigation.

The proteomic analysis also identified proteins associated with epidermal growth factor receptor (EGFR)- and tumor-associated calcium signal transducer 2 (TROP2) signalling, both of which intersect with MAPK/ERK and PI3K/Akt cascades involved in cell proliferation and survival [41]. EGFR signalling has been reported to attenuate OA progression and promote cartilage preservation [42], and TROP2 has been linked to IGF-I-related pathways including Wnt/β-catenin [43]. In the absence of direct validation, these observations should be regarded as hypothesis-generating and are not further interpreted here.

Finally, components of the ESCRT machinery, including PDCD6IP (ALIX), were identified among the regulated proteins. Given the role of ESCRT complexes in endosomal trafficking, receptor sorting, and membrane remodelling [44–46], their enrichment may reflect increased vesicular trafficking activity in IGF-I-transduced cells, potentially linked to the higher biosynthetic demand associated with chondrogenic differentiation.

Taken together, many of the pathways identified in this study converge on processes required to sustain the high biosynthetic demands of chondrogenesis, including energy metabolism, protein processing, intracellular trafficking and stress adaptation. This suggests that the effects of IGF-I extend beyond direct stimulation of matrix synthesis and may involve broader mechanisms that facilitate the acquisition and maintenance of a chondrogenic phenotype in OA-derived BM-MSCs.

This study has several strengths. To our knowledge, this is the first proteomic characterization of the molecular response to IGF-I gene therapy in OA-derived BM-MSCs undergoing chondrogenic differentiation, addressing a gap in the understanding of the molecular mechanisms underlying rAAV-mediated gene therapy for OA. From a technical standpoint, the use of a state-of-the-art DIA-PASEF proteomic platform enabled deep proteome coverage, and a rigorous statistical framework was applied for differential expression analysis. Furthermore, the orthogonal validation of selected proteins by IHC and IF strengthens the reliability of the proteomic findings. Nevertheless, certain limitations should be considered when interpreting these findings. Standard immunophenotypic characterization of the isolated BM-MSCs was not performed for the donors included in the present study due to the limited cell yield obtained from the available bone marrow aspirates. The number of donor-derived BM-MSC populations used for proteomic analysis was low (*n* = 3), which constrains statistical power and the generalizability of the results, despite the generation of five independent micromass cultures per condition. The IHC and IF analyses were performed using BM-MSCs from a single additional donor and should therefore be interpreted as supportive evidence of the proteomic findings rather than as validation of their reproducibility across donors. Functional validation of the identified pathways was beyond the scope of this work, and the proteomic enrichment of metabolism-related pathways should be interpreted as hypothesis-generating observations pending functional confirmation. Additionally, BM-MSCs derived from OA patients may exhibit altered biological properties compared with cells from healthy donors, which may limit extrapolation to a non-pathological context. As an entirely in vitro study, the present findings cannot be directly extrapolated to conclusions about cartilage regeneration or therapeutic efficacy in OA. Previous studies have demonstrated the regenerative potential of rAAV-mediated IGF-I delivery in vivo [13, 47, 48], but further validation in more advanced three-dimensional models and in vivo systems will be required, acknowledging the additional translational challenges associated with rAAV delivery.

## Conclusion

This study provides, to our knowledge, the first proteomic characterization of the effects of rAAV-mediated IGF-I gene therapy during the chondrogenic differentiation of OA-derived BM-MSCs, contributing novel molecular insight into a promising therapeutic strategy for cartilage regeneration. IGF-I delivery was associated with broad proteomic changes involving metabolic and protein processing pathways, consistent with enhanced biosynthetic activity, as well as increased cartilage-specific matrix deposition. Orthogonal validation by IHC and IF confirmed the IGF-I-associated upregulation of XPO1 and β-catenin, supporting the involvement of nucleocytoplasmic transport and Wnt signalling in the cellular response to IGF-I. These findings provide insight into the molecular effects of IGF-I gene transfer during in vitro chondrogenic differentiation and highlight pathways of potential relevance to cartilage repair. Validation in larger cohorts and complementary experimental models will be required to establish their functional relevance and therapeutic implications in vivo.

## Supplementary Information

Supplementary Material 1: Supplementary Table 1. Proteins identified in the proteome of BM-MSCs undergoing chondrogenesis. Cells from OA patients were transduced with IGF-I or lacZ via rAAV. Provided as an Excel table, showing only those proteins detected with ≥ 2 unique peptides.

Supplementary Material 2: Supplementary Table 2. Exploratory analysis of protein detection patterns in BM-MSCs transduced with rAAV-IGF-I compared with lacZ controls. Provided as an Excel table, listing proteins with *p*-value ≤ 0.05 based on chi-squared analysis.

Supplementary Material 3: Supplementary Table 3. Proteins quantitatively altered in BM-MSCs transduced with IGF-I, compared with lacZ. Significant differential expression was considered a fold change ≥ 1.5, together with an adjusted *p* ≤ 0.05. Provided as an Excel table.

Supplementary Material 4: Supplementary Table 4. Results from the pathway and process enrichment analysis. This analysis was performed on the 112 significant proteins. Provided as an Excel table.

## Data Availability

The mass spectrometry proteomics raw datasets generated and analysed during the current study have been deposited to the ProteomeXchange Consortium via the PRIDE partner repository with the dataset identifier PXD074694.

## Abbreviations

AAV: Adeno–associated virus
ACN: Acetonitrile
AKT: Protein kinase B
ALDH3A2: Fatty aldehyde dehydrogenase
APOE: Apolipoprotein E
BGN: Biglycan
BM: MSC–Human bone marrow–derived adherent stromal cells
C1QBP: Complement component 1 Q subcomponent–binding protein
CMV: IE–Cytomegalovirus immediate–early promoter
COL: II–Type II collagen
β: Catenin–Catenin beta 1
DAPI: 4′,6–Diamidino–2–phenylindole
DIA: Data–independent acquisition
DIA: NN–Data Independent Acquisition by Neural Networks
DMEM: Dulbecco’s Modified Eagle Medium
DTT: Dithiothreitol
ECM: Extracellular matrix
EGF: Epidermal growth factor
EGFR: Epidermal growth factor receptor
ER: Endoplasmic reticulum
ERK: Extracellular signal–regulated kinase
ESCRT: Endosomal sorting complex required for transport
FA: Formic acid
FBS: Fetal bovine serum
FC: Fold change
FDR: False discovery rate
GAG: Glycosaminoglycan
GLMP: Glycosylated lysosomal membrane protein
GO: Gene Ontology
GPC: Glypican
IA: Iodoacetamide
IF: Immunofluorescence
IGF: I–Insulin–like growth factor I
IHC: Immunohistochemistry
KEGG: Kyoto Encyclopedia of Genes and Genomes
LC: Liquid chromatography
LC: MS/MS–Liquid chromatography–tandem mass spectrometry
lacZ: β–galactosidase–encoding control gene
MAPK: Mitogen–activated protein kinase
MCODE: Molecular Complex Detection
MOI: Multiplicity of infection
MPRIP: Myosin phosphatase Rho–interacting protein
MRPL30: Mitochondrial ribosomal protein L30
MRPS10: Mitochondrial ribosomal protein S10
MRPS25: Mitochondrial ribosomal protein S25
MSC: Mesenchymal stem cell
MS: Mass spectrometry
MV: Missing values
NFKB1: Nuclear factor NF–kappa–B p105 subunit
OA: Osteoarthritis
OGT: O–linkedβ–N–acetylglucosamine transferase
PASEF: Parallel Accumulation–Serial Fragmentation
PCA: Principal component analysis
PDCD6IP (ALIX): Programmed cell death 6–interacting protein
PG: Proteoglycan
PI3K: Phosphatidylinositol 3–kinase
PPI: Protein–protein interaction
rAAV: Recombinant adeno–associated virus
RT: Room temperature
SAR1A: Small COPII coat GTPase
SD: Standard deviation
SDS: PAGE–Sodium dodecyl sulfate–polyacrylamide gel electrophoresis
SIRT1: Sirtuin 1
TFA: Trifluoroacetic acid
TGF: β3–Transforming growth factor beta 3
TIMS: Trapped ion mobility spectrometry
TOF: Time–of–flight
TROP2: Tumor–associated calcium signal transducer 2
XPO1 (CRM1): Exportin 1
YRDC: Threonyl carbamoyl–AMP synthase
